# The Sardinian Puzzle: Concentration of Major Psychoses and Suicide in the Same Sub-Regions Across One Century

**DOI:** 10.2174/1745017901713010246

**Published:** 2017-11-30

**Authors:** Alberto Bocchetta, Francesco Traccis

**Affiliations:** Department of Biomedical Sciences, Section of Neurosciences and Clinical Pharmacology, University of Cagliari, Cagliari, Italy

**Keywords:** Mood disorders, Bipolar and related disorders, Schizophrenia spectrum and other psychotic disorders, Suicide, Hospital, Psychiatric, Altitude, Population Size, Sardinia

## Abstract

**Background:**

Sardinia, the second largest Mediterranean island has long been considered a privileged observatory for the study of several medical conditions. The peculiar epidemiology of mood disorders and suicide across Sardinian sub-regions has long intrigued clinicians and researchers.

**Objective:**

The principal aim of the present study was to test whether the geographical distribution of suicides committed in Sardinian over the last three decades are comparable with the geographical origin of patients hospitalized up to half a century ago.

**Method:**

The distribution of the municipalities of origin of the patients hospitalized in Sardinia between 1901 and 1964 for schizophrenia, bipolar disorder, and depression was reanalyzed and compared with the distribution of municipalities where suicides were committed between 1980 and 2013. Data were also analyzed by the altitude above the sea level and by the population size of the municipalities.

**Results:**

There was a significant variation of hospitalization and suicide rates across Sardinian sub-regions. The sub-regions of origin of the patients hospitalized for schizophrenia and bipolar disorder correlated with each other (*P* = 0.047). Both hospitalizations and suicides were more incident in municipalities with a higher altitude and a smaller population size. The incidence of hospitalizations and suicides correlated significantly with each other both at the municipality (*P* = 1.86 x 10^-7^) and at the sub-region level (*P* = 1.71 x 10^-7^).

**Conclusion:**

The present study confirms the peculiar geographical distribution of major psychoses and suicide in Sardinia. The two phenomena appear to have been correlated for as long as one century.

## 1 INTRODUCTION

Sardinia, the second largest Mediterranean island with a current population of 1.65 million, has long been considered a privileged observatory for the study of medical conditions. Islands are often intriguing for epidemiologists and geneticists because they may represent geographically isolated pockets with unique populations. Just to give an example regarding the psychiatric field, the series of studies started in the 1930s by Erik Strömgren in the island of Bornholm, Denmark are among the most frequently cited epidemiological genetic works in the history of psychiatry [1]. In his doctoral thesis, Strömgren provided a complete census of psychoses existing in Bornholm in 1935, and the census was repeated half a century later [2].

The peculiar epidemiology of mood disorders and suicide across Sardinian sub-regions has long intrigued clinicians and researchers. The first attempts to survey the incidence of mental disorders in Sardinia municipalities were published in the 1960s [3, 4]. In the 1970s, Athanasios Koukopoulos noted that a strikingly high number of his patients with bipolar disorder came from one small village in Sardinia. After first consulting with Strömgren, Koukopoulos tried to undertake an epidemiological study there, but the progress in this unfunded community survey was limited [5].

Our group started to study bipolar and related disorders in Sardinia in the 1980s. The high prevalence of G6PD deficiency in Sardinia prompted us to survey G6PD activity in our population of patients with the initial aim of identifying pedigrees for an X-linkage study [6]. Unexpectedly, we found an excess of G6PD deficiency in the subgroups of patients with bipolar spectrum disorders [7-10]. We suggested that the uneven distribution of G6PD deficiency throughout Sardinia, depending on the once malaria endemicity, may explain in part why bipolar syndromes appear to be enriched in some areas, as already suggested by the two 1960s surveys of psychiatric hospitalizations [3, 4].

In the meantime, we surveyed also heterozygous β-thalassemia as a phenotypic marker to be used in a linkage study with chromosome 11p15, which was popular in psychiatric genetics at that time. Unexpectedly, similarly to G6PD deficiency, we found excessive proportions of heterozygous β-thalassemia among subgroups of patients with bipolar spectrum disorders, especially those with psychotic symptoms [7, 11, 12]. One possible explanation of such disproportions is that the two genetic conditions typical of Mediterranean areas have pleiotropic effects and play a role in psychiatric syndromes, even though a bias due to geographical stratification cannot be ruled out. With the advent of DNA molecular markers, our group undertook several genetic association studies of candidate loci for bipolar illness, but results were not substantial [13-22].

Over the last decades, we have also investigated clinical characteristics of bipolar spectrum disorders and suicide behavior within families of Sardinian patients [23, 24]. More recently, we focused again on the geographical distribution of mental illness across Sardinian sub-regions. For example, we found higher suicide mortality rates in municipalities whose lithium content in stream sediments is lower (submitted for publication).

Given the known relationship between suicide and major psychiatric illness, the aim of the present study was to test whether the geographical distribution of suicides committed in Sardinia over the last three decades may be comparable with the geographical origin of patients hospitalized in the two Sardinian mental hospitals up to half a century ago.

## 2 METHODS

### 2.1 Reappraisal of Data on Former Psychiatric Hospitalizations in Sardinia (1901-1964)

The aforementioned survey by Camba and Rudas [4] regarded all clinical records of patients hospitalized between 1901 and 1964 in the two provincial mental hospital of Cagliari and Sassari, which were instituted in 1901. Patients were classified by birthplace. Sardinian municipalities in 1964 amounted to 335. The paper provided geographical charts showing the incidence of hospitalizations for various disorders across the municipalities of origin. In addition, the cumulative incidence rates were provided regarding the 33 so-called historical regions of Sardinia. The latter subdivision is still in use for some purposes, even if not officially corresponding to the current administrative regions. It reflects the ancient subdivisions dating back to medieval times, mostly based on the absence of geographical barriers, the relative language homogeneity, and the tendency towards endogamy. The survey studied 16,305 hospital records, which can be considered a very representative sample of severe psychiatric disorders in Sardinia in the first half of the XX century.

In the original survey, the rates for single municipalities were calculated taking into account the average number of residents during the period under study. The overall Sardinian population between 1901 and 1964 averaged 1,043,452. The diagnoses were based on classifications in force at that time in Italy. The authors considered the following nine nosographic classes (corresponding current diagnoses are shown in parentheses): 1) schizophrenic syndromes; 2) paranoia and delusional syndromes; 3) oligophrenic syndromes (intellectual disability); 4) epilepsy; 5) alcohol-related disorders and toxic psychoses; 6) dysthymias (bipolar disorder); 7) psychoneurotic syndromes (major depression); 8) progressive paralysis (general paresis or paralytic dementia due to neurosyphilis); 9) dementia syndromes. It must be noted that infectious brain diseases and epilepsy might led to psychiatric hospitalizations because specific medications were not available yet.

### 2.2 Data on Suicides (1980-2013)

The Italian National Institute of Statistic (ISTAT) provides the database of all causes of death from medical certification sorted by codes according to the World Health Organization International Classification of Disease (ICD-9 and ICD-10). We extracted data on suicides occurred in Sardinian municipalities during the periods 1980-2003 and 2006-2013. Data regarding single municipalities were not available for the years 2004 and 2005. Suicides corresponded to ICD-9 categories E950-E959 (in the 1980-2003 period), and to ICD-10 categories X60-X84 (in the 2006-2013 period).

Demographic data were extracted from the Italian National Institute of Statistics—ISTAT web site (http://demo.istat.it/index.html), in which population data were obtained from Population Register Offices of each Italian municipality. Data available from the register are estimates in the intercensal period (ten years) and inferred by elaborating data concerning the last census and the demographic flows, such as births and deaths, in the decades considered.

We calculated the cumulative incidence of suicide per 1000 residents for each municipality according to the average residential population over the period from 1980 to 2013.

Sardinia currently consists of 377 municipalities whose population ranges from one hundred to 150,000 inhabitants. Many hamlets have become independent since the 1965 survey on psychiatric hospitalizations, when municipalities amounted to 335 [4]. The principal variation in population size over the last decades has regarded larger cities and coastal towns whose population has increased, whereas the majority of smaller villages has maintained a similar population size. The overall Sardinian population size has varied from 1,594,000 (1981 census) to 1,639,362 (2011 census).

To avoid bias potentially associated with outliers, we also calculated cumulative suicide incidence in Sardinian sub-regions, similar to those used in the 1965 survey on psychiatric hospitalizations [4]. We chose 39 sub-regions based on a slight modification of the plan for the reassessment of the best administrative subdivisions published in 2006 in a deliberation by the Sardinian government (Regione Autonoma della Sardegna: Deliberazione Giunta Regionale, 15 dicembre 2006, n. 52/2).

### 2.3 Population Size of Municipalities and Altitude

We calculated the cumulative incidence of hospitalizations for schizophrenia, bipolar disorder, and depression between 1901 and 1964 and the cumulative suicide incidence with a view to the population size of the municipalities and their altitude above the sea level.

### 2.4 Correlation Between Hospitalizations (1901-1964) and Suicides (1980-2013)

We compared the incidence of hospitalizations for the three principal psychiatric disorders (schizophrenia, bipolar disorder, depression) between 1901 and 1964 and the incidence of suicides recorded between 1980 and 2013, using two levels of geographical subdivisions (municipalities and historical sub-regions).

## 3 RESULTS

### 3.1 Reappraisal of Data on Former Psychiatric Hospitalizations in Sardinia (1901-1964) with New Calculations

According to the original survey [4], the cumulative incidence of individuals hospitalized for any diagnosis in the two Sardinian mental hospitals between 1901 and 1964 was 15.6 per 1000 residents. The incidence rates per 1000 residents for the single diagnostic subgroups were the following: schizophrenia = 5.37; delusional syndromes = 0.37; intellectual disability = 1.86; epilepsy = 0.89; alcohol-related disorders and toxic psychoses = 1.08; bipolar disorder = 1.93; major depression = 1.37; neurosyphilis = 0.55; dementia = 2.18.

The distribution by area of origin was similar between schizophrenia, bipolar disorder, and depression. There was an “epicenter” of municipalities with higher incidence for such disorders in the south east area of Sardinia. The distribution regarding the remaining diagnostic subgroups did not reveal any particular pattern. The epicenter was graphically evident in the charts included in the original publication depicting the 33 historical sub-regions.

According to our new calculations, the overall cumulative incidence of hospitalizations for schizophrenia, bipolar disorder, and depression between 1901 and 1964 in Sardinia was 8.7 per 1000 residents (95% CI = 8.5-8.8). These represented 56% of all the hospitalizations recorded. There was a threefold variation between the birthplace sub-region with the highest rate (Barbagia di Seulo = 15.8 per 1000 residents; 95% CI = 12.9-18.9) and the birthplace sub-region with the lowest rate (Sulcis = 5.3 per 1000 residents; 95% CI = 4.7-5.9).

The hospitalization rates for schizophrenia and bipolar disorder across the 33 historical sub-regions correlated with each other (*R* = 0.35; *P* = 0.047).

The rates of hospitalizations for schizophrenia, bipolar disorder, and depression were lower for the individuals born in towns with more than 10,000 residents (that represented 29% of all residents) (7.1 per 1000 residents; 95% CI = 6.8-7.4) compared to the individuals born in smaller municipalities (9.3 per 1000 residents; 95% CI = 9.1-9.5).

There was a positive correlation between the hospitalization rates for schizophrenia, bipolar disorder, and depression and the altitude of the 335 municipalities (Multiple *R* = 0.38; *P* = 1.11 x 10^-12^).

### 3.2 Data on Suicides Recorded Between 1980 and 2013

The overall number of suicides committed in the 377 Sardinian municipalities during the study period (1980-2013) amounted to 4967, corresponding to a cumulative incidence of 3.0 per 1000 residents (95% CI = 2.9-3.1).

There was a five-fold variation between the sub-region with the highest rate (Barbagia di Seulo = 9.5 per 1000 residents; 95% CI = 6.9-12.7) and the sub-region with the lowest rate (Baronie = 1.8 per 1000 residents; 95% CI = 1.4-2.3).

Suicide rates were lower for the individuals born in towns with more than 10,000 residents (that represented 52% of all residents) (2.6 per 1000 residents; 95% CI = 2.5-2.7) compared with the individuals born in smaller municipalities (3.4 per 1000 residents; 95% CI = 3.3-3.6).

There was a positive correlation between the suicide rates and the altitude of the 377 municipalities (Multiple *R* = 0.26; *P* = 3.58 x 10^-7^).

### 3.3 Correlation Between Hospitalizations (1901-1964) and Suicides (1980-2013).

**Fig. (1):**
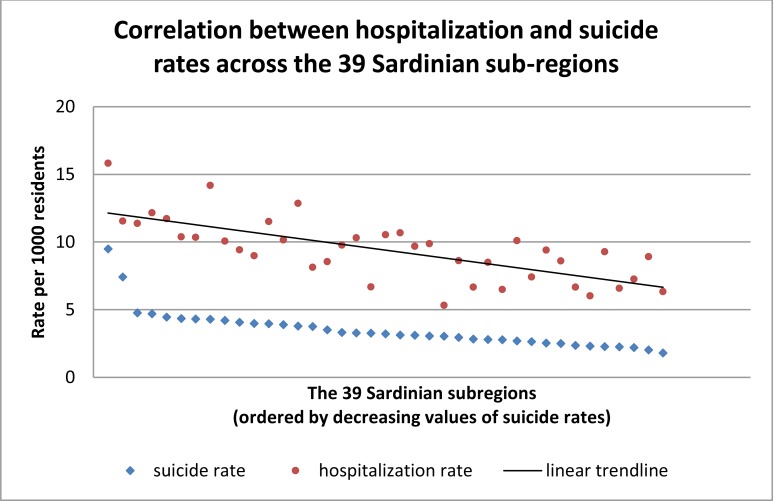
For graphical purposes, data were ordered by decreasing values of suicide rates.

When examining the 335 municipalities existing in Sardinia in 1964, the incidence of hospitalizations recorded between 1901 and 1964 for the three principal psychiatric disorders (schizophrenia, bipolar disorder, and depression) and the incidence of suicides recorded between 1980 and 2013 correlated significantly (*R* = 0.28; *P* = 1.86 x 10^-7^). Similar results were obtained by examining the 39 Sardinian historical sub-regions (*R* = 0.73; *P* = 1.71 x 10^-7^) (Fig. **1**).

## 4 DISCUSSION

The availability of data on past hospitalizations and recent suicides divided by municipality has allowed the principal interesting observation from the present study that major psychoses and suicide have had similar distributions within Sardinian sub-regions across one century. A secondary observation was that the correlation with altitude and the rural/urban ratio were similar between hospitalizations and suicides.

We must underscore that both mental illness and suicide are complex phenomena with many underlying factors. Their shared geographical distribution, as found in the present study, may depend on sociocultural, genetic and/or environmental aspects, but it can provide an initial step aiming at identifying new potential tiles in the Sardinian puzzle. Recent studies have confirmed that isolated populations can provide interesting hints in the psychiatric field, as was for example the case of the traditional Amish community [25]. The Amish Study of Major Affective Disorder (ASMAD), initiated in the 1970s has tracked several multi-generation pedigrees with high prevalence of bipolar spectrum disorders [26]. All suicides for a 100-year period (1880 to 1980) were ascertained [27]. The majority (92%) of suicide cases were diagnosed with a major affective disorder and were situated in multigenerational families with heavy loading for bipolar, unipolar, and other affective-spectrum illnesses.

The population originating from some Sardinian sub-regions can be considered as isolated as the Amish community, and may also provide interesting data. Interestingly, a recent study of the Amish families combining microsatellite and high-density single nucleotide polymorphism (SNP) genotypes with whole-genome sequence data implicated dozens of rare alleles that may interact to determine risk for bipolar disorder [28]. Other data from the Amish study suggest potential roles of functional variation of neuronal potassium channels or copy number variants which may be contributing factors in the phenotypic presentation and heterogeneity of mental illness [29, 30]. Thus, it has become evident, as also suggested from other genetic studies that single genetic variants may result in a wide range of psychopathological presentations [31-33]. The latter observation challenges the validity of current nosographic classifications and may even be consistent with the results from the Sardinian survey of hospitalizations showing similar distributions between schizophrenia and mood disorders.

The incidence rates of hospitalizations for schizophrenia appear at least two times greater than those of bipolar disorders, that is at odds with current nosology, but it must be noted that the presence of psychotic symptoms in patients with affective episodes often resulted in a diagnosis of schizophrenia up to the advent of current criteria (at least up to 1980, when DSM-III began to be used also in Italy) and to the introduction of lithium therapy in the 1970s.

The comparable geographical distribution between hospitalizations and suicide rates found in the present study is consistent with the evidence that mental illness is present among >90% of suicides [34, 35]. The lifetime risk of suicide is estimated to be 4% in patients with mood disorders [36], 8% in people with bipolar disorder [37, 38], and 5% in people with schizophrenia [39].

To date, the interpretation of the reasons of the variable geographical distribution of mental illness and suicides have largely been influenced by the perspective of the XIX century French pioneer sociologist Émile Durkheim. For example, in his book “*Suicide*” published in 1897 [40], Durkheim focused on family and social factors, including marital status, religion, employment, and social integration. One of the principal issues has been the rural/urban ratio in suicide rates.

In the present study, we found that both hospitalizations and suicides were more incident in small villages. Similar patterns are being observed in recent studies of other populations [41, 42].

Another interesting aspect shared with studies from other countries is the positive correlation between suicide and altitude above the sea level, that has been attributed to the effects of metabolic stress associated with mild hypoxia in individuals with mood disorders [43-45].

Besides the risk factors shared with other populations, our hypothesis is that some genetic and/or environmental peculiarities may contribute to the geographical distribution of mental illness and suicide in Sardinia. For example, our series of the aforementioned studies suggested that G6PD deficiency and heterozygous β-thalassemia, two hematological conditions typical of the Mediterranean area, can have pleiotropic effects, including a potential role in bipolar-spectrum disorders [7-12]. With regard to suicide, we also suggested a role of low cholesterol concentrations, which may depend on either genetic factors and/or specifically Mediterranean dietary habits [46]. More recently, we suggested that comorbidity with thyroid autoimmunity, which is very prevalent in Sardinia [47] may be associated with psychiatric manifestations including suicidality [48, 49]. With particular regard to the geographic distribution across Sardinian sub-regions, we would like to mention our recent finding that the concentration of lithium in stream sediments correlates inversely with suicide mortality (manuscript in preparation). A protective effect of naturally occurring lithium against suicide has already been suggested by research groups from several countries studying lithium content in drinking water (for review see [50]). The relationship between lithium content in drinking water and suicide may also have complex interactions with altitude [51], which is was in turn associated with hospitalization and suicide rates in the present study.

## CONCLUSION

The present study confirms prior suggestions that there is a peculiar geographical distribution of major psychoses and suicide in Sardinia. The two phenomena appear to have been correlated for as long as one century. These data can be the base for further studies aiming at unraveling the complex pathogenesis of major psychoses and suicide. One potential approach, similar to that already used in the Amish study [29], would be to search for candidate genetic variants carried by affected members of families originating from specific Sardinian isolates.
